# Gut microbiota at the crossroads of cocaine use and binge eating disorders

**DOI:** 10.3389/fnbeh.2026.1841613

**Published:** 2026-07-17

**Authors:** Jeanne Tyrode, Victor Mathis, Katia Befort

**Affiliations:** 1 Université de Strasbourg, CNRS, LNCA (Laboratoire de Neurosciences Cognitives et Adaptatives), UMR 7364, Strasbourg, France; 2 INSERM UMR-S 1329, Strasbourg Translational Neuroscience and Psychiatry, Centre de Recherche en Biomédecine de Strasbourg, Université de Strasbourg (UNISTRA), Strasbourg, France

**Keywords:** addiction, binge eating disorder, cocaine use disorder, gut-brain axis, microbiota

## Abstract

Binge eating disorder (BED) and cocaine use disorder (CUD) are two major public health concerns characterized by impaired control over consumption and significant impacts on physical and mental health. BED, the most prevalent eating disorder, involves recurrent episodes of excessive intake of highly palatable foods, while CUD is marked by compulsive cocaine use. Both disorders involve neurotransmitters dysregulation prompting research into their neurobiological overlap. Emerging clinical and preclinical research highlights the microbiome as a critical modulator of behavior and mental health. This review critically evaluates the role of the microbiome in BED and CUD, focusing on preclinical and clinical evidence on microbial composition and functional pathways, including microbiome-targeted interventions. We highlight gaps in the current literature that warrant further investigation. Microbial alterations may serve as one potential biomarker to clarify shared or distinct mechanisms in the progression and maintenance of the pathophysiology of these disorders.

## Graphical abstract

**Figure:**
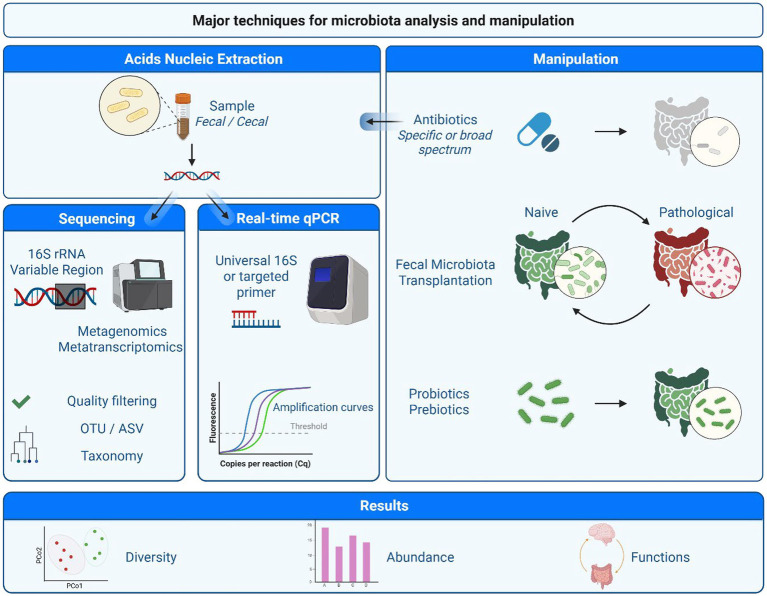
Overview of methods for assessing and manipulating gut microbiome composition and function. This toolbox outlines the most commonly used methods for evaluating microbiome composition and function, both under baseline conditions and following experimental interventions. (Left panel) After nucleic acid extraction from fecal or cecal samples, a range of techniques -including quantitative PCR (qPCR), 16S rRNA sequencing, metagenomics, and metatranscriptomics- have revolutionized our understanding of gut microbial ecosystems. These approaches enable the identification of microbial species, their functional roles, and interactions with the host, offering a comprehensive view of microbial diversity, abundance, and activity. Among these techniques, 16S ribosomal RNA (rRNA) sequencing stands out as a widely used method for identifying and comparing bacterial communities within samples by targeting highly conserved regions of ribosomal genes. Metagenomics analyzes the entire genetic material from microbial communities, offering insights into their genetic potential. Metatranscriptomics focuses on sequencing RNA transcripts, revealing the active gene expression profiles, clarifying the functional roles of microbial populations. (Right Panel) Strategies for actively manipulating the gut microbiome. Antibiotic treatments can be applied to deplete microbial populations at specific time points, either broadly or targeting specific bacteria, followed by the potential reintroduction of selected bacterial species to study their contributions. Efficacy of depletion is assessed via qPCR or 16S rRNA sequencing. Other approaches include fecal microbiota transplantation (FMT), where fecal material from a donor is transferred into the gastrointestinal tract of a recipient to directly alter microbiome composition, enabling causal investigations of microbial roles in health and disease. Finally, the use of prebiotics and probiotics offers a promising avenue for modulating gut microbial composition and function, with potential applications in both research and clinical settings. (Bottom panel) These analyses allow to describe diversity, abundance and functions of bacterial populations. Created in https://BioRender.com.

## 1 Introduction

Eating disorders are severe psychiatric conditions that profoundly impair both physical and mental health in the world (Treasure et al., 2020). Among them, binge eating disorder (BED) is the most prevalent, affecting 1.4% of the adult population (Kessler et al., 2013). BED was formally recognized as a distinct diagnosis in the Diagnostic and Statistical Manual of Mental Disorders (DSM-5) in 2013 and later included in the International Classification of Diseases (ICD-11) in 2019 (Giel et al., 2022). It is characterized by recurrent episodes of consuming large amounts of highly palatable food, typically rich in sugar and fat, within a short time and in the absence of compensatory behaviors. It is frequently associated with obesity and psychiatric comorbidities (Udo and Grilo, 2018, 2019). A core feature of BED is the subjective loss of control over eating (Colles et al., 2008; Oswald et al., 2011; Moshe et al., 2017), which has contributed to ongoing debates regarding the concept of “food addiction,” given its parallels with core symptoms observed in substance use disorders (Fletcher and Kenny, 2018; Gordon et al., 2018; Hough et al., 2026).

Indeed, this conceptual link has led researchers to draw parallels between BED and substance use disorders (SUD), another major public health concern worldwide. SUD are defined by a set of behavioral, cognitive, and physiological criterion, notably including excessive consumption and impaired control over intake, as outlined in the DSM-5 and ICD-11 classifications (Hasin et al., 2013; The Lancet, 2019). A substantial body of research has investigated similarities and differences between eating disorders and SUDs at the level of brain circuitry and neurotransmitter systems (Schreiber et al., 2013). Cocaine use disorder (CUD), one of the most prevalent forms of substance abuse worldwide (European Monitoring Centre for Drugs and Drug Addiction, 2024), shares some mechanisms with BED, with imbalance in the mesocorticolimbic reward circuit, DA and DA-independent regulations, as well as synaptic plasticity (Wang et al., 2011; Jones and Fordahl, 2021; Leenaerts et al., 2022; Di Chiara and Imperato, 1988; Hernandez and Hoebel, 1988; Kalivas and Duffy, 1990). Recent work has particularly pointed to the endocannabinoid system as a common regulated neuromodulator system in reward processing, both in CUD and BED (Spanagel, 2020; Bourdy and Befort, 2023). Nevertheless, central mechanisms of these multifactorial disorders are complex and more research is needed to better comprehend their pathophysiology.

In recent years, growing interest in the gut microbiome (Cryan and Dinan, 2012) has opened new avenues for understanding psychiatric disorders, highlighting the importance of peripheral systems, particularly the gut–brain axis, in modulating behavior and mental health (for recent reviews see Meckel and Kiraly, 2019; Borkent et al., 2022; Li et al., 2022; Cammann et al., 2023; Hashimoto, 2023; Shukla and Hsu, 2025). The microbiome has indeed emerged as a promising source of candidate biological markers that may help future diagnostic research. Several clinical and preclinical studies have recently explored the potential dysbiosis observed in CUD and recent research has also developed in BED. Therefore, we decided in this review, to focus our attention on the role of the microbiome in BED and CUD to help clarify potential shared or distinct mechanisms in these two pathologies.

## 2 Microbiome and binge eating disorder

Composition of the gut microbiome is strongly influenced by dietary changes and stress, which are the two main factors involved in the onset and maintenance of BED (Guo and Xiong, 2024). In addition, several hypotheses have proposed that the gut microbiota plays a role in BED, including dysbiosis or specific microbial profiles that may influence the patients feeding behaviors. Consistent with these hypotheses, Breton et al. provided the first clinical evidence identifying gut microbiota alterations in BED, showing increased plasma levels of ClpB, a protein produced by commensal *Escherichia coli*, specifically in women with BED. ClpB mimics *α*-melanocyte-stimulating hormone (α-MSH), an anorexigenic neuropeptide involved in food intake regulation (Breton et al., 2016). Recent advancements in methodologies investigating the microbiome, notably sequencing techniques and bio-informatic analysis providing insights onto microbial diversity and abundance (see Toolbox), helped to uncover the potential role of microbiota in eating disorders.

Main findings in clinical (a) and preclinical (b) studies investigating the alteration of microbiota in BED are summarized in Table 1. As an example for studying microbiota in food-related disorders, a recent study examined the microbiome composition of patients with BED and found that salivary microbial diversity was significantly lower than normal-weight healthy controls. More precisely, this study revealed an enrichment of the class Bacilli in patients suffering from BED (Mercante et al., 2024). Noteworthy, we only found one study focusing on salivary microbiome following BED. Indeed, numerous other clinical studies have focused on gut microbiota. For example, Castellini et al. investigated microbiota composition according to eating-disorder behavioral profiles, distinguishing patients with binge-purging related disorders (including patients with BED) from patients with restrictive eating disorders, and controls. The binge-purging group showed a higher relative abundance of the phylum Bacteroidetes and an enrichment of the genus *Prevotella* (Castellini et al., 2023). Another study investigated the gut microbiota composition in obese patients. It compared individuals with and without BED revealed an increased relative abundance of the genus *Anaerostipes* and a decreased abundance of the genera *Intestinimonas, Akkermansia* and *Desulfovibrio* specifically in patients with BED (Leyrolle et al., 2021). Among them, *Anaerostipes* and *Intestinimonas* both produce butyrate. Butyrate is a short chain fatty acid (SCFA), a key player for the maintenance of gut function. In particular, *Intestinimonas butyriciproducens* has proven beneficial for host metabolism in the context of obesity (Rampanelli et al., 2025). Therefore, the impact of such opposite regulation for these two genera appears difficult to interpret. As pointed out by the authors, more research is needed to improve current knowledge of the relationship between co-existing bacteria (Leyrolle et al., 2021). Members of the genus *Desulfovibrio* are sulfate-reducing bacteria present in the human gastrointestinal tract that produce hydrogen sulfide gas (H_2_S). This microbial metabolite has been linked to the development of several diseases when present in excess (Singh et al., 2023). Notably, H_2_S also exerts important physiological functions at low concentrations, including roles in cellular signaling, mucosal protection, and regulation of inflammation. On the other hand, *Akkermansia muciniphila* plays a beneficial role in several conditions, including diabetes, obesity, aging, cancer, and metabolic syndrome (Mo et al., 2024), and has been widely studied for these protective effects and its association with improved insulin sensitivity (Macchione et al., 2019). Based on this evidence, a reduced abundance of *Akkermansia* may be involved in the mechanisms underlying binge-eating related phenotypes. Interestingly, both *Akkermansia* and *Desulfovibrio* were also decreased in a study investigating patients suffering from obesity and presenting a high score in food addiction (FA; Dong et al., 2020), suggesting that these bacteria may impact compulsive behaviors. Together, these results highlight a strong alteration in the gut microbiota that could contribute to the progression or maintenance of the disorder.

**Table 1A:** BED and microbiota.

a. Clinical study					
Ref	Sex	Age	Pathology/Condition	Methods (sample)	Results **Group**/*Analysis*
Leyrolle et al. (2021)	F, M	18–65 yo	Obesity with BED (*N* = 38) Obesity without BED (*N* = 53)	16S rRNA Seq *(fecal)*	**Obesity with BED vs w/o BED** *PLS-DA (VIP score > 1,5)* Increase: *Anaerostipes* Decrease: *Akkermansia, Desulfovibrio, Intestinimonas*
Dong et al. (2020)	F	18–50 yo	Obesity with Food Addiction (*N* = 17) Obesity without Food Addiction (*N* = 34)	16S rRNA Seq *(fecal)*	**Obesity with food addiction vs w/o food addiction** *DESEq2* Negative association *Eubacterium biforme, Bacteroides plebeius, Sutterella, Catenibacterium, Desulfovibrio, Bacteroides, Akkermansia muciniphila, Ruminococcus* Positive association *Odoribacter*, Ruminococcaceae, Barnesiellaceae, *Alistipes massiliensis* and *Megamonas* *PLS-DA* *Streptococcus*: the largest contribution
Castellini et al. (2023)	F	18–43 yo	AN, BN, BED with Binge-Purging (*N* = 22) AN Restricting (*N* = 9)	16S rRNA Seq *(fecal)*	**Binge-purging** *Beta Diversity (Bray-Curtis)* Differentiation between Binge-Purging group and the other samples *LEfSe (LDA score threshold = 3,5)* Increase: Bacteroidetes and *Prevotella*
Igudesman et al. (2023)	F, M	18–45 yo	BED or BN with laxative use (*N* = 31) BED or BN without laxative use (*N* = 234)	16S rRNA Seq *(fecal)*	**BED or BN with laxative use vs w/o laxative use** *Alpha diversity (Chao-1, Faith, Shannon)* Decrease *Normalized abundance* Decrease: *Eubacterium ventriosum, Alistipes, Bilophila*, and GCA900066575
Mercante et al. (2024)	F	16–60 yo	BED (*N* = 20) BN (*N* = 17)	16S rRNA Seq *(saliva)*	**BED and BN vs healthy controls** *Alpha diversity (Shannon)* Decrease *Beta diversity (Bray Curtis)* BED and BN are similar but differ from healthy controls *Relative abundance* Increase: Bacilli

b. Prelinical study							
Ref	Species	Sex, Age	Pathology exposure	Micobiota manipulation	Palatable items	Methods (sample)	Results **Group**/*analysis*
Kaakoush et al. (2017)	Rat	M, 6–8 wo	Cycle cafeteria: 16 weeks [3 d caf; 4 d standard chow] (*N* = 11)	/	Cafeteria diet	16S rRNA Seq *(fecal)*	**Cycle cafeteria vs control** *Relative abundance* Increase: Coriobacteriaceae, Lachnospiraceae, Porphyromonadaceae, *Blautia* and *Collinsella* Decrease: Ruminococcaceae, Enterobacteriaceae, *Escherichia* *PICRUSt* Increase: Carbohydrate digestion and absorption Decrease: Arachidonis acid, Alpha-linolenic acid, Ketone bodies and flavone synthesis, D-arginine and nucleotide metabolism, Geraniol degradation
Leigh et al. (2020)	Rat	F, 4–5 months	Cycle cafeteria: 7 weeks [3 d caf; 4 d standard chow] (*N* = 11–12)	/	Cafeteria diet	16S rRNA Seq *(fecal)*	**Cycle cafeteria vs Continuous cafeteria** *LefSe and DeSeq2* Increase: Porphyromonadaceae unclassified_OTU35 Decrease: Coprobacter_OTU66 **Cycle cafeteria vs control** *PICRUSt* Decrease: Lipid metabolism, Flavone and Flavonol and Flavonoid biosynthesis
Fan et al. (2023)	Mouse	F, 6–16 wo	*Overeating disorder* OD: 3x [4 d food restriction; 2 d normal diet + Oreo; 4 d normal diet; 1 d stress (foot shock)] (*N* = 5)	/	Oreo cookies	16S rRNA Seq *(fecal)*	**Overeating disorder vs control** *Alpha Diversity (Chao1, Shannon)* Decrease *Beta diversity (Bray-Curtis)* Differentiation between groups *Relative abundance* Increase: Bacteroidaceae, Lachnospiraceae Decrease: Lactobacillaceae, Ruminococcaceae *LEfSe* Loss: *Lactobacillus*, Ruminococcaceae-UCG014 Increase: *Bacteroides, Roseburia* and *Alistipes*
			
-ODs + FMT from control -Control + FMT from ODs (*N* = 5)		/	**ODs + FMT from control vs ODs** Almost completely prevented overeating behaviors **Control + FMT from ODs vs control** No obvious overeating behavior was detected
Agustí et al. (2021)	Rat	M, Adult (170– 200 g)	*Food addiction* Intermittent fasting (IF): 18 d [12 h fasting, 12 h HSD] (*N* = 15)	/	Sucrose solution 10%	16S rRNA Seq *(cecal)*	**Intermittent fasting vs control** *Beta diversity (Bray Curtis)* Differentiation between IF and control *LDA (score ≥ 3.0)* Decrease: *Muribaculum* spp., *Eubacterium* spp., *Prevotella copri*
			
IF + treated with *B. uniformis* (IF+B) (*N* = 15)		/	**Intermittent fasting + B uniformis vs IF** *LDA (score ≥ 3.0)* Increase: *A. muciniphila, C. minuta*, *and F. umbilicata* *B. uniformis* protected IF animals against the loss of *Muribaculum* spp., *B. uniformis* reverses anxiety-like behavior and reduces caloric intake during binge eating
Demangeat et al. (2025)	Mouse	F, 8 wo	HFHS: 10 d [2 h every 2 d]	HFHS + ABX in the drinking water: [3 d antifungals + 1 w antibiotics] (*N* = 8)	HFHS		**HFHS + ABX vs HFHS** Increase: cumulated food intake during the intermittent access

**Table 1B:** Cocaine and microbiota.

a. Clinical study					
Ref	Sex	Age	Pathology/Condition	Methods (sample)	Results **Group**/*Analysis*
Gerace et al. (2023)	F, M	Mean: 43.8 yo	Cocaine users (*N* = 58)	16S rRNA Seq *(fecal and saliva)*	**Cocaine users vs healthy controls** *Alpha diversity (Observed, Shannon in fecal and Pielou’s evenness in saliva)* Decrease: in fecal and saliva samples *Beta diversity (Bray-Curtis)* Differentiation between healthy controls and cocaine users (for fecal and saliva) *DESeq2:* Fecal Increase: Erysipelotrichaceae, *Blautia* spp., *Collinsella* spp., *Dorea* spp., *Romboutsia* spp. *and Streptococcus* spp. Decrease: Christensenellaceae, Desulfovibrionaceae, Lachnospiraceae, *Alistipes* spp., *Bacteroides* spp.,and *Barnesiella* spp. Saliva Increase: *Rothia* spp., *Staphylococcus* spp. and *Treponema* spp. Decrease: *Haemophilus* spp., *Neisseria* spp. *and Porphyromonas* spp. *PICRUSt2* (LEfSe, LDA > 2) Fecal Increase: purine degradation, pyrimidine, amino acids and phospholipid biosynthesis and pentose phosphate pathway Saliva Increase: isoleucine, valine synthesis, purine and pyramidine biosynthesis and degradation
Volpe et al. (2014)	F, M	20–60 yo	Cocaine users (with or w/o HIV) (*N* = 14) Non–cocaine users (with or w/o HIV) (*N* = 18)	16S rRNA Seq *(fecal)*	**Cocaine users vs non-cocaine users** *Relative abundance* Increase: Bacteroidetes
Fu et al. (2022)	F, M	18–55 yo	Cocaine users (*N* = 8)	16S rRNA Seq *(saliva)*	**Cocaine users vs non-cocaine users** *Alpha Diversity (Simpson, Shannon)* Decrease *Relative abundance* Increase: Firmicutes, *Streptococcus* Decrease: Proteobacteria, *Actinobacillus, Campylobacter, Fusobacterium, Haemophilus, Mannheimia, Neisseria, and Porphyromonas*

b. Prelinical study						
Ref	Species	Sex, Age	Exposure	Microbiota manipulation	Methods (sample)	Results **Group**/*analysis*
Chivero et al. (2019)	Mouse	M, 8–10 wo	1 i.p./d/7 d cocaine (20 mg/kg) (*N* = 10)	/	16S rRNA Seq *(fecal and colon)*	**Cocaine vs saline** *Beta diversity (UPGMA Weighted UniFrac)* Differentiation between cocaine vs. saline-administred mice *Relative abundance* Colon Decrease: *Mucispirillum, Butyricicoccus, Pseudoflavonifractor and unclassified Ruminococcaceae* Increase: *Barnesiella, unclassified members of Porphyromonadaceae, Bacteroidales unclassified,* Proteobacteria*, Erysipelotrichaceae unclassified, Streptococcus, Sphingomonas, Pasteurellaceae unclassified, Alistipes, Desulfovibrionaceae unclassified* Fecal Decrease: *Lachnospiracea incertae sedis, Pseudoflavonifractor and Streptophyta* Increase: *Turicibacter, Alistipes, Odoribacter, unclassified members of Porphyromonadaceae* and Proteobacteria
Angoa-Pérez et al. (2023)	Mouse	F, 18–25 g	1 i.p/d/10 d -Cocaine Hydrochloride (20 mg/kg) -Cocaine Methiodide (26 mg/kg) -MDPV (2 mg/kg) (*N* = 6–7/group)	/	16S rRNA Seq *(cecal)*	*Beta Diversity (Bray Curtis and Jaccard)* Differentiation between all the groups **Cocaine Hydrochloride (HCl) vs others groups** *BLAST and LEfSe (LDA score threshold > 3.0)* Increase: *Akkermansia muciniphila, Muribaculum intestinale, Muribaculaceae bacterium* and 3 strains of *Lactobacillus* **Cocaine Methiodide (MI)** *BLAST and LEfSe (LDA score threshold > 3.0) vs others groups* Increase: *Duncaniella muris, Muribaculum* sp. *TLL-A4, Turicimonas muris* *Relative abundance vs control* Decrease: Ruminococcaceae **Cocaine HCl and cocaine MI vs control** *Relative abundance* Increase: Verucomicrobia, Akkermansiaceae Decrease: Deferribacteraceae, Desulfovibrionaceae, Rikenellaceae **MDPV** *BLAST and LEfSe vs others group* Enriched: *Lactobacillus faecis* *Relative abundance vs control* Decrease: Verrucomicrobia, Desulfovibrionaceae, Akkermansiaceae Increase: Lachnospiraceae *PICRUSt2* Random forest Cofactors and vitamins metabolism, lipid metabolism and carbohydrate metabolism are differentiate between 3 drugs and controls
Scorza et al. (2019)	Rat	M, Adults (250–310 g)	Drug volatilization 10 min/d/14 d -Cocaine (25 mg/d) -Caffeine (25 mg/d) -Phenacetin (25 mg/d) (*N* = 3/group)	/	16S rRNA Seq *(fecal)*	**Cocaine vs control** *Alpha diversity (Shannon)* Decrease *Beta diversity (Weighted Unifrac)* Cocaine and phenacetin-treated rats were different compared to control rats *SIMPER* Increase: Lachnospiraceae (*Lachnoclostridium*), Prevotellaceae, and Peptostreptococcaceae (*Peptoclostridium* spp.) *Relative abundance* Decrease: Ruminococcaceae *PICRUSt* Increase: butyrate synthesis Decrease: aromatic amino acid decarboxylase **Phenacetin vs control** *SIMPER* Increase: Lactobacillaceae (*Lactobacillus* spp.), Veillonellaceae (*Quinella* spp.), Ruminococcaceae *Relative abundance* Increase: Firmicutes, *Bacilli, Clostridia*, Bacteroidales S24–7, Lachnospiraceae, *Lactobacilli* Decrease: Bacteroidetes, Bacteroidia, Prevotellaceae *PICRUSt* Decrease: Phosphate butyryl transferase, aromatic amino acid decarboxylase **Cocaine and Phenacetin vs control** *SIMPER* Decrease: Desulfovibrionaceae (*Desulfovibrio*), Spirochaetaceae (*Treponema* sp.)
Li et al. (2023)	Mouse	F, M 6, 12 months	16 groups: 2 strains (WT/iTat) × 6/12 months × M/F × 2 treatments Cocaine (i.p. 30 mg/kg/d/14 d) Saline (*N* = 12/group)	/	16S rRNA Seq *(large intestine)*	**Cocaine-WT** M, 6 months *Beta diversity (Bray-Curtis)* Differentiation between groups *Relative abundance* vs. saline-WT Increase: Tannerllaceae F; 12 months *Alpha diversity and Beta diversity* Differentiation between groups (Shannon and Bray Curtis respectively) *Relative abundance* vs. saline-WT Increase: Bacteroidaceae, Muribaculaceae Decrease: Desulfovibrionaceae, Ruminococcaceae, Clostridiales_vadinBB60_group *Tax4Fun* vs. saline-WT Increase: lipid metabolism **Saline-iTat mutant mice** F, 6 months *Relative abundance* Increase: Alteromonadaceae in iTat, reversed by cocaine exposure
Chesworth et al. (2024)	Mouse	M, 8 wo	Cocaine-induced CPP (i.p. 20 mg/kg) (*N* = 12)	/	16S rRNA Seq *(fecal)*	**Cocaine** *Alpha diversity (Shannon, Observed Features)* Increase with persistent effects *Correlations* CPP score for cocaine is positively correlated with abundance of Actinobacteria
Binh Tran et al. (2023b)	Mouse	F, M >12 wo	Cocaine IVSA (*N* = 128) Acquisition (18 d max): 1 mg/kg/inf ACQ = 5 sessions > 10 inf; otherwise FACQ (*N* = 100)	/	16S rRNA Seq *(fecal)*	**Acquired (ACQ) vs failed (FACQ)** *Beta diversity (Bray Curtis)* Differentiation between ACQ and FACQ *Relative abundance* Increase: *Barnesiella, Ruminococcus, and Robinsoniella*, Bacteroidetes Decrease: *Clostridium IV*, Firmicutes ACQ males Decrease: *Lactobacillus* ACQ females Decrease: *Blautia* *Correlation* Positive correlation between the doses administered during the acquisition phase and relative abundances of *Robinsoniella* and *Clostridium IV* *PICRUSt2* Gut-brain modules different between ACQ and FACQ: GABA synthesis, glutamate and propionate metabolism, dopamine and GHB degradation *mWGS* Gut-brain modules different between ACQ and FACQ: glutamate metabolism, methionine, acetate, isovaleric acid and S-adenosylmethionine synthesis Upregulated pathways: glutamate degradation, peptidoglycan and heme biosynthesis Downregulated pathway: fatty acid salvage
Binh Tran et al. (2023a)	Mouse	F, M 8 wo	High cocaine-responding strain (CC004) 19 d cocaine sensitization (i.p. 10 mg/kg) (*N* = 6)	/	16S rRNA Seq *(fecal)*	**Sensitization to cocaine-CC004 strain** *Beta diversity (Bray-Curtis)* Differentiation between pre- and post-cocaine *Relative abundance pre- vs post-cocaine* Increase: *Barnesiella* and *unclassified_Coriobacteriaceae* *PICRUSt2 pre- vs post-cocaine* Decrease: gut brain modules related to tryptophan synthesis, glutamine metabolism, glutamate and GABA synthesis Increase: Vitamin K2 synthesis
			
CC004 -ABX in drinking water (1 w initiation + during protocol) + 8 d cocaine sensitization (i.p. 5 mg/kg) (*N* = 24) -ABX in drinking water + IVSA: (*N* = 5) Acquisition (18 d max): 1 mg/kg/inf ACQ = 5 sessions > 10 inf Dose response (max 5 d/dose): 1.0; 0.32; 0.1; 0.032 mg/kg/inf	/	**Sensitization to cocaine-CC004 strain with ABX** *Locomotor counts* Increase: cocaine-sensitization in female vs. w/o ABX *IVSA* Increase: infusions during IVSA dose–response curve vs. w/o ABX
García-Cabrerizo et al. (2023)	Mouse	M 6 wo	/	ABX (in drinking water during 21 d) + Cocaine-induced CPP (i.p. 10 mg/kg) (*N* = 12)	/	**Cocaine + ABX** *CPP* Decrease: CPP score vs. cocaine w/o ABX Increase: CPP score compare to Saline ABX
Winters et al. (2025)	Mouse	M, F 7–8 wo	/	Cocaine-induced CPP (i.p. 20 mg/kg) -Germ Free (GF) -CONV-Raised -CONV-GF -ABX (oral gavage 1 x d/21 d) (*N* = 12–32/group)	/	**Germ free (GF) vs conventional (CONV-R)** *Locomotor activity* Decrease: cocaine-evoked psychomotor activation *CPP* Decrease: CPP score **CONV-GF vs GF** *Locomotor activity* Restoration of locomotor responses to cocaine *CPP* Restoration of CPP score **ABX vs w/o ABX** *Locomotor activity* Decrease: cocaine-evoked psychomotor activation *CPP* Decrease: CPP score
Kiraly et al. (2016)	Mouse	M 7 wo	/	Cocaine-induced CPP (i.p. 5 or 10 mg/kg) -ABX (in drinking water 7–10 d + duration of protocol) (*N* = 8) -SCFA (in the drinking water) (*N* = 5–22) -ABX (3 d) + SCFA (*N* = 5–22)	/	**ABX vs w/o ABX** *Locomotor sensitization* No difference (cocaine 10 mg/kg) Increase (cocaine 5 mg/kg) *CPP* No difference on CPP score 10 mg/kg Induction of CPP (cocaine 5 mg/kg) **SCFA vs w/o ABX** *CPP* No difference on CPP score (5 mg/kg) **SCFA + ABX vs w/o ABX** *CPP* Reverse ABX effect: No difference on CPP score (5 mg/kg)
Cuesta et al. (2022)	Mouse	M 11–13 wo	/	-Orally infected: 1 × 10^9^ CFU *C. rodentium* WT + Cocaine: 1x/d/5d (i.p. 15 mg/kg) or saline (*N* = 6–9) -ABX: (3 d orally treated) + Orally infected with y-Proteobacteria (1 d): *-C.rodentium* ΔescN 1 × 10^9^ CFU -E. Coli 1 × 10^10^ CFU + Cocaine-induced CPP (i.p. 5 mg/kg) (*N* = 6–9)	/	**Cocaine vs saline** Increase: *C. rodentium* colonization **Colonization effect** *Locomotor activity* Increase: cocaine-induced sensitization *CPP* Induction of cocaine-CPP
Dave et al. (2025)	Mouse	F 7 wo	/	ABX (drinking water/14 d) + Cocaine-induced CPP (i.p. 2.5, 5 or 10 mg/kg) (*N* = 9–11)	/	**ABX vs w/o ABX** *Locomotor sensitization* Decrease: locomotor sensitization (10 mg/kg) *CPP* Increase: CPP score (5 mg/kg) Decrease: CPP score (10 mg/kg)
Meckel et al. (2024)	Rat	M Adult (300–320 g)	/	IVSA ACQ (food restriction): (0.8 mg/kg/inf) + Threshold testing or Abstinence 21 d -ABX (drinking water/14 d + duration of protocol) (*N* = 9–11) -ABX + SCFA (drinking water/14 d + duration of protocol) (*N* = 9–11)	/	**ABX vs w/o ABX** *IVSA* Increase: motivation for low dose cocaine on a within-session threshold task Increase: cue-induced cocaine-seeking following prolonged abstinence **ABX + SCFA repletion** Reverse ABX-effects on cocaine-seeking after abstinence

ABX: antibiotic; ACQ: acquired; AN: anorexia nervosa; ASV: amplicon sequence variants; BED: binge eating disorder; BN: bulimia nervosa; CONV-R: conventionally raised; CPP: conditioned place preference; CUD: cocaine use disorder; d: day; ED: eating disorder; F: female; FA: food addiction; FACQ: failed to acquire; FMT: fecal microbiota transplantation; GF: germ free; h: hour; HCl: hydrochloride; HFD: high fat diet; HFHSD: high fat high sugar diet; HSD: High sugar diet; ICD-11: international classification of diseases; IF: intermittent fasting; Inf: Infusion; i.p.: intraperitoneal; IVSA: Intravenous self-administration; M: Male; MDPV: methylenedioxypyrovalerone; MI: methiodide; OD: overeating disorder; OTU: operational taxonomic unit; rRNA Seq: 16S ribosomal RNA sequencing; SCFA: short-chain fatty acid; SUD: substance use disorder; w: week; wo: week-old; w/o: without; WT: Wild type; yo: year-old; blue lines are listing manipulations of the microbiota.

Contrasting the clinical work, only a few studies have investigated gut microbiota composition and function in animal models of binge-eating. Preclinical BED models aim to replicate essential features of the human condition, combining environmental stressors with controlled dietary manipulations, such as cycles of restriction/refeeding and intermittent access to palatable foods and beverages (Brown and James, 2023; Awad et al., 2024; Guo and Xiong, 2024). Such models represent powerful tools to investigate specific mechanisms developed in this pathology, using binge-eating conditions in isolation from other comorbid factors. Four studies investigating the impact of such eating behavior on the gut microbiota have used this type of experimental paradigms leading to binge-like phenotype (see Table 1, section b). First, Fan et al. implemented a model of overeating disorder (OD) in female mice consisting of three cycles of food restriction (4 days), followed by a normal diet together with Oreo cookies (HFD, 2 days) and 4 days of normal diet without fat, with an acute stressor applied at the end of each cycle. This paradigm revealed a binge-like phenotype, which resulted in changes in the relative abundance of several bacterial families, including an increase in Bacteroidaceae and Lachnospiraceae, and a decrease in Lactobacillaceae and Ruminococcaceae. At the genus level, LEfSe analysis revealed a decrease in *Lactobacillus* and *Ruminococcaceae-UCG-014*, and an increase in *Bacteroides*, *Roseburia* and *Alistipes* in OD mice (Fan et al., 2023). Another study characterized gut microbiota composition in male rats using a FA model using intermittent fasting (12 h) and intermittent access to a high-sucrose diet (HSD; 12 h) for 18 days. This protocol resulted in a binge-like phenotype, and revealed a decrease in the genera *Muribaculum* spp. and *Eubacterium* spp., as well as in the species *Prevotella copri* (Agustí et al., 2021). Finally, two studies revealed that intermittent access to a cafeteria diet 3 days per week, resulting in binge-like phenotype, induced different microbiota changes in male (Kaakoush et al., 2017) than in female rats (Leigh et al., 2020). Notably, these studies did not highlight common alterations between humans and rodent models. Such a discrepancy may reflect species-specific differences, but may also arise from the type and diversity of palatable food (fat and/or sugar), frequency and duration of access used in these animal models; human studies being by nature more difficult to control than animal studies. It also points to other factors like sex that are critical for examining the role of the microbiome in binge eating.

Beyond describing microbiota alterations, some studies have also experimentally manipulated the microbiota in order to test its potential causal behavioral contributions (see Toolbox, and blue lines in Tables 1, 2). A recent study used a mouse model of BED based on intermittent access to a high-fat high-sugar diet (HFHSD; 2 h every 2 days for 10 days). Half of the mice received antibiotics (ABX) in their drinking water for one week, leading to microbiota depletion and increased cumulative food intake during the intermittent access (Demangeat et al., 2025), supporting a modulatory role of the microbiome in eating behaviors. In a similar manner, fecal microbiota transplantation (FMT) from control mice to an OD mouse model almost completely prevented overeating behavior. Interestingly, FMT from OD mice to control mice did not induce overeating behavior (Fan et al., 2023). This suggests that restoring a “healthy” microbiome can alleviate or even prevent pathological conditions, whereas microbiota alterations alone may be insufficient to trigger such conditions, consistent with the multifactorial nature of eating disorders. In the FA model, *Bacteroides uniformis* administered orally on a daily basis before the beginning of the fasting period, reduced caloric intake during binge eating, decreased anxiety-like behavior as well as reversed the fasting-induced microbiota changes and increased the abundance of species linked to healthy metabolotypes, highlighting the interplay between microbiota species (Agustí et al., 2021). Interestingly, the administration of *Bacteroides uniformis* led to an increase in the abundance of *Akkermansia muciniphila*, which is consistent with previous findings showing a decrease in *Akkermansia* in binge-like phenotypes (Agustí et al., 2021).

Although informative, these results from preclinical studies are highly variable, likely due to the limited number of studies but also the differences in study designs and exposure conditions. Nonetheless, the findings reveal a close relationship between BED and the gut microbiota. The composition of the gut microbiome is directly shaped by BED, as well as by dietary patterns. Importantly, these studies demonstrate that targeted manipulation of the microbiota can influence feeding behaviors, highlighting its potential as a therapeutic strategy to improve BED symptoms. However, further well-controlled studies, particularly in humans where it is difficult to avoid any heterogeneity, are needed to confirm these effects and to establish their clinical relevance.

## 3 Microbiome and cocaine use disorder

The mechanism of action and the consequences of cocaine intake on brain functions has been studied extensively (Kalivas, 2004; Nestler, 2005; Koob and Volkow, 2016). Briefly, by blocking dopamine, norepinephrine and serotonin transporters, cocaine artificially maintains high brain levels of monoamines (Ritz et al., 1987), responsible for the euphoric effects of the drug. Nonetheless, cocaine also has peripheral effects, including altering cardiac rhythmicity (Schwartz et al., 2010) and body temperature (Callaway and Clark, 1994; Crandall et al., 2002). In accordance with these peripheral aspects, several recent studies have demonstrated that cocaine intake could influence microbiota and participate in CUD (Meckel and Kiraly, 2019). Table 2 summarizes main findings in clinical (a) and preclinic (b) studies investigating the alteration of microbiota following cocaine exposure. In preclinical studies, various experimental paradigms, such as conditioned place preference (CPP) assessing reinforcing properties of drugs and self-administration (SA) assessing voluntary consumption and motivational aspects, were used to evaluate the impact of cocaine exposure (intraperitoneal, systemic or vapor administration) on locomotor activity and reward-related behaviors.

Two recent studies with cocaine users reported a significant reduction in salivary microbial alpha diversity compared to non-users (Fu et al., 2022; Gerace et al., 2023). Although sparse, several clinical studies suggest that gut microbiota is altered in patients suffering from CUD (Volpe et al., 2014; Gerace et al., 2023). Indeed, a study from Volpe and colleagues revealed a higher abundance of Bacteroidetes in cocaine users (Volpe et al., 2014). Recently, Gerace and colleagues described a clear dysbiosis in the fecal microbiota of patients with CUD compared to healthy controls characterized by a decreased alpha diversity and modification of the abundances of several taxa. At the genus level, patients with CUD showed higher fecal abundances of *Blautia, Collinsella, Dorea, Romboutsia and Streptococcus,* but reduced levels of *Alistipes, Bacteroides, and Barnesiella* (Gerace et al., 2023).

Similar microbiome alterations have been reported in preclinical experiments with several studies showing that repeated cocaine administration is sufficient to provoke a clear dysbiosis in mice (Chivero et al., 2019; Binh Tran et al., 2023a; Li et al., 2023). Chivero and colleagues demonstrated that such dysbiosis is associated with alterations in the gut barrier integrity and function, notably with increased intestinal permeability, creating a favorable inflammatory loop that might participate in CUD development or maintenance (Chivero et al., 2019). Similar alterations in gut microbiota composition have been observed in rats exposed to volatilized cocaine (Scorza et al., 2019). Interestingly, a study investigating the role of microbiota on cocaine intake in IVSA paradigm, reported differences in gut microbiota composition between mice that acquired and those that failed to acquire the self-administration, suggesting a potential role of the microbiota in addiction susceptibility (Binh Tran et al., 2023b).

In the gut-brain relationship, the origin of the dysbiosis is unknown: does it result from a direct effect of cocaine in the gut, or indirectly after its central effect? Evidence from a recent study suggests that a peripheral mechanism may be involved, as cocaine hydrochloride and cocaine methiodide, that does not cross the blood–brain barrier, have similar effects on the gut microbiota composition (Angoa-Pérez et al., 2023). Overall, these preclinical studies highlight common cocaine-induced changes in gut microbiota. In particular, the families Erysipelotrichaceae (Chivero et al., 2019; Gerace et al., 2023) and Muribaculaceae (Angoa-Pérez et al., 2023; Li et al., 2023) as well as the genus *Streptococcus* (Chivero et al., 2019; Gerace et al., 2023) are increased following cocaine exposure. In contrast, several studies report decreased abundances of the families Desulfovibrionaceae and Ruminococcaceae (Chivero et al., 2019; Scorza et al., 2019; Angoa-Pérez et al., 2023; Gerace et al., 2023; Li et al., 2023). Noticeably, similar observations were performed in patients with drug-addictive profile, with higher abundance of *Streptococcus* (alcohol use disorder and smokers), and reduced abundance of Ruminococcaceae (alcohol and opioid use disorder and smokers; Borrego-Ruiz and Borrego, 2025), highlighting potential involvement of microbiota in the development or maintenance of other SUDs.

Using different manipulations in rodents (see Toolbox), recent studies have confirmed the critical relationship between microbiota composition, bacteria-derived metabolite and cocaine-related behaviors. For instance, Meckel and colleagues demonstrated that antibiotic-induced microbiota depletion enhances motivation for low doses of cocaine and increases cue-induced cocaine-seeking following prolonged abstinence in rats (Meckel et al., 2024). Similar findings were reported in male (Kiraly et al., 2016) and female (Dave et al., 2025) mice, where microbiome depletion increased or induced cocaine CPP at a low dose (5 mg/kg). However, antibiotics treatments reduced cocaine CPP at higher doses (10 mg/kg; García-Cabrerizo et al., 2023; Dave et al., 2025). In addition, studies using germ-free or antibiotic treated mice reported reduced psychomotor responses to higher cocaine doses (10–20 mg/kg; Dave et al., 2025; Winters et al., 2025), whereas other studies observed increased locomotor activity following lower cocaine doses (5 mg/kg) in microbiota-depleted animals (Kiraly et al., 2016; Binh Tran et al., 2023a, 2023b).

Altogether, these findings shed light on the close interplay between gut microbiota composition and cocaine-related behaviors. As for BED, data obtained from patients may differ from those obtained in preclinical setting. The latter are strictly controlled while the former might have other conditions associated with CUD, notably food-related behaviors that are altered by cocaine intake (Ersche et al., 2013). Nonetheless, together, these studies not only suggest that direct modification of the microbiota by the drug might contribute to CUD symptoms but also that targeting the microbiota might represent a novel therapeutic approach for patients with CUD.

## 4 Unraveling common microbiome alterations in BED and CUD

To compare microbiota regulation between the two pathologies, we considered only bacterial taxa consistently reported as increased or decreased across examined studies within a given pathology (BED or CUD). Indeed, some contradictory findings across studies made it difficult to draw reliable conclusions regarding the direction of microbiota alterations (see details in Tables).

**Figure 1:**
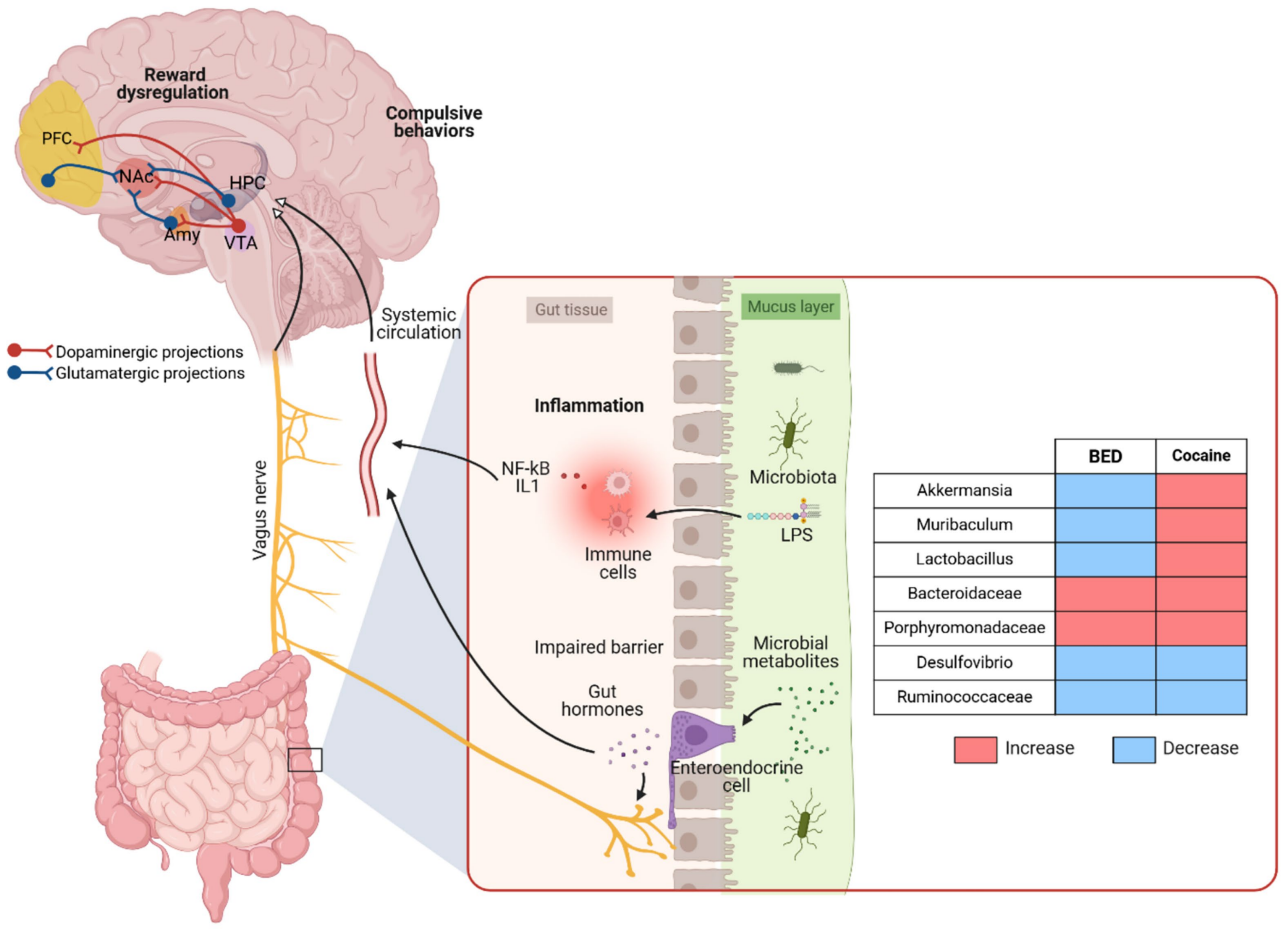
Proposed mechanisms linking gut microbiota alterations to neurobehavioral changes in BED and CUD. Gut–brain communication occurs through four major pathways: immune signaling, vagal signaling, microbial metabolites, and hormonal regulation, which are described in detail in the following reviews (Guo and Xiong, 2024; Lorsch and Liddle, 2026; Samulėnaitė et al., 2026). The heatmap summarizes bacterial taxa that are either decreased (light blue) or increased (light red) in both disorders. Only bacterial taxa regulated in both disorders are shown. These alterations may exacerbate the gut-brain dysregulation. LPS can induce low-grade inflammation leading to increased intestinal permeability (Cani et al., 2007), thereby promoting the release of pro-inflammatory mediators such as NF-κB and IL-1β into the bloodstream (Chivero et al., 2019). These inflammatory mediators can subsequently contribute to systemic inflammation and potential neuroinflammation. In parallel, microbiota-derived metabolites regulate hormonal release by stimulating enteroendocrine cells (Chao et al., 2025). Together, inflammatory mediators and hormones may act on the brain through the vagus nerve and systemic circulation (white arrows). Altogether, these hormonal and inflammatory processes will modulate the reward brain circuits (reward dysregulation) and contribute to the behavioral alterations (compulsive behaviors) observed in BED and CUD. Whether microbial regulation directly causes behavioral adaptations still remains to be clarified (PFC: prefrontal cortex; NAc: nucleus accumbens; HPC: hippocampus; Amy: amygdala; VTA: ventral tegmental area; LPS: lipopolysaccharide dysregulation). Created in https://BioRender.com.

Overall, both divergent and convergent regulations between CUD and BED were described (see summary in Figure 1). Several studies on BED have reported a decrease in beneficial bacterial taxa, including *Akkermansia* (Leyrolle et al., 2021), *Muribaculum* (Agustí et al., 2021), and *Lactobacillus* (Fan et al., 2023) whereas these taxa were increased in cocaine-related study (Angoa-Pérez et al., 2023). Specific species like *Akkermansia muciniphila* and *Muribaculum intestinale* exert protective effects by strengthening intestinal barrier integrity, promoting the production of SCFAs, and reducing neuroinflammation (Feng et al., 2025; Wang et al., 2026). In addition, these bacteria act on depressive-like phenotype: *Akkermansia muciniphila* restored hypothalamic–pituitary–adrenal axis function and abnormal variations in depression-related molecules (corticosterone, dopamine, and brain-derived neurotrophic factor (BDNF) in a chronic restraint stress-induced depression model (Ding et al., 2021); *Muribaculum intestinale* has also been associated with increased BDNF levels and improvements in depressive-like behaviors (Cao et al., 2025b). Similarly, *Lactobacillus rhamnosus* modulates the expression of GABA receptors in several brain regions, including cortical areas, the amygdala, and the hippocampus. It also reduces stress- and anxiety-related behaviors through mechanisms dependent on vagal nerve signaling (Bravo et al., 2011). Taken together, these divergent microbial signatures suggest distinct roles of gut microbiota in the progression of CUD and BED, whereby enrichment of beneficial taxa in CUD may reflect compensatory responses to cocaine-induced neurobiological alterations, whereas their depletion in BED could contribute to the persistence of pathological eating behaviors.

Interestingly, our review also highlights common alterations in CUD and BED. Increased abundance of *Porphyromonadaceae* (Kaakoush et al., 2017; Chivero et al., 2019; Leigh et al., 2020) *and Bacteroidaceae* (Fan et al., 2023; Li et al., 2023) *have been observed in association with both BED and CUD*. The bacterial family Porphyromonadaceae showed increased abundance in both anxiety and depression (Cao et al., 2025a). *Bacteroidaceae is a highly abundant bacterial family within the gut microbiota.* Administration of a particular species of this family, *Bacteroides uniformis*, in a FA model with binge-like phenotype, reduced anxiety-like behaviors. These are associated with alterations in dopamine, serotonin, and noradrenaline levels in the NAc, as well as changes in D1 and D2 dopamine receptor expression in the PFC and the intestine (Agustí et al., 2021). Another species, *Bacteroides fragilis*, possesses lipopolysaccharides (LPS) at its surface (Lukiw, 2016). LPS can induce low-grade inflammation by stimulating the secretion of pro-inflammatory mediators from host cells and increasing intestinal permeability, thereby promoting systemic inflammatory responses (Cani et al., 2007). These pro-inflammatory molecules may enter the bloodstream and contribute to neuroinflammatory processes. An increased abundance of genera belonging to the Lachnospiraceae family is consistently observed in BED- (*Anaerostipes, Roseburia, Blautia*; Kaakoush et al., 2017; Leyrolle et al., 2021; Fan et al., 2023) and cocaine- (*Blautia*, *Lachnoclostridium*; Scorza et al., 2019; Gerace et al., 2023) related studies. Members of Lachnospiraceae family have also been positively associated with decreased D2 mRNA receptor expression in the dorsal striatum in a rat model of alcohol use disorder (Jadhav et al., 2018). Conversely, several studies report a decreased abundance in *Desulfovibrio* (Scorza et al., 2019; Leyrolle et al., 2021) *and Ruminococcaceae* (Kaakoush et al., 2017; Chivero et al., 2019; Scorza et al., 2019; Angoa-Pérez et al., 2023; Fan et al., 2023; Li et al., 2023) following cocaine intake and BED. *Desulfovibrio* has been associated with obesity and is known to produce both H_2_S and LPS (Zhou et al., 2024), suggesting a potential contribution to inflammatory and metabolic dysregulation. Interestingly, Jadhav and colleagues have shown that Ruminococcaceae has also been associated with decreased D2 mRNA receptor expression in the dorsal striatum in a rat model of alcohol use disorder (Jadhav et al., 2018), suggesting a link between this family and addictive-like behaviors. Altogether, these overlapping microbial alterations raise the possibility that certain microbial signatures may be similar across disorders, reflecting shared biological pathways underlying compulsive behaviors and reward dysregulation.

Figure 1 provides a summary of the distinct pathways connecting modifications in gut microbiota to neurobehavioral phenotypes in BED and CUD. Communication between the gut and brain via immune signaling, vagal pathways, microbial metabolites, and hormonal regulation, underpins this relationship (see recent reviews for more details: Guo and Xiong, 2024; Lorsch and Liddle, 2026; Samulėnaitė et al., 2026). Microbial shifts observed may trigger a cascade: LPS-induced low-grade inflammation increases intestinal permeability, releasing pro-inflammatory mediators into circulation, while microbiota-derived metabolites stimulate enteroendocrine cells to release hormones. Together, these inflammatory and hormonal signals act on the brain via the vagus nerve and systemic circulation, ultimately modulating reward pathways, contributing to the compulsive behaviors observed in BED and CUD. Whether a direct causal relationship exists between microbial regulations and behavioral adaptations remains to be clarified.

It is worth noting that many of the microbial taxa identified across BED and CUD have also been implicated in broader metabolic, sleep, inflammatory, stress-related and psychiatric conditions (Molina-Torres et al., 2019; Acevedo-Román et al., 2024; Dos Santos and Galiè, 2024; Espinosa et al., 2026). This convergence underscores the central role of the gut microbiota in human health and disease. The recurrence of these taxa across diverse disorders further suggests they may contribute to shared pathophysiological mechanisms and so transcend traditional diagnostics. On the other hand, opposite microbial shifts between BED and CUD mirror patterns in other psychiatric disorders. For instance, Akkermansia is decreased in autism but increased in schizophrenia. Similarly, Lactobacillus is reduced in anorexia nervosa yet elevated in autism, schizophrenia, and ADHD. These observations emphasize the need to consider global microbial signatures across psychiatric conditions when identifying candidate biomarkers. Understanding both the convergent and divergent microbiota signatures associated with BED and CUD will prove essential for providing a critical foundation for the development of personalized microbiota-based therapeutic strategies. Furthermore, identifying shared and disorder-specific microbial characteristics can yield valuable insights into the ongoing debate surrounding food addiction and participate in developing new diagnostic resources.

## 5 Conclusion and perspectives

While food acts as a natural reinforcer that engages reward-related brain systems, drugs of abuse pharmacologically destabilize these same circuits, producing supraphysiological effects (García-Cabrerizo et al., 2021). Despite the fundamentally distinct nature of food consumption and drug use, their associated disorders, such as BED and CUD, share common clinical features, including impulsivity, compulsive and impaired control of intake. This overlap in clinical features suggests the involvement of shared mechanisms in their pathology, although these mechanisms remain to be fully elucidated. In this review, we describe that BED and CUD indeed share common gut microbiota signatures such as those involving the bacterial species listed above, but also demonstrate opposite regulatory patterns for some specific taxa, contributing to the specificity of each pathology. However, several limitations must be acknowledged. First concerning the microbiota analysis, technical variations such as the nature of the samples, the sequencing targets (e.g., variable regions) or the data template (OTU vs. ASV) provide challenges to direct comparisons across studies. Additionally, the taxonomic resolution (e.g., phylum vs. genus) may introduce inconsistencies in interpreting bacterial data. Second, a key concern lies in the challenges posed by the complexity of the pathologies, as well as the potential comorbidities that may interfere with their progression. In human research, BED is often confounded by comorbidities like obesity or other psychiatric conditions that may influence feeding patterns, which may obscure specific pathological mechanisms. Also, substance or medication use is not always assessed in patient cohort and might influence the microbiome and so the conclusion of each study. Additionally, clinical studies also often describe small sample size, which contribute to the complexity of the interpretation. Meanwhile, BED models are powerful tools to decipher bingeing behavior, but remain limited. Indeed, palatable food items serve as critical variable factors in disease development, yet their diversity, associated with the multitude of paradigms used, with varying outcomes, potentially influence the results and interpretation. These clinical and preclinical observations could also apply to CUD studies. These gaps underscore the need for further research into gut-brain interactions, particularly the role of fat and sugar in feeding behaviors. More study designs should also account for sex differences, which appear pivotal in adaptive responses. Furthermore, emerging evidence of shared microbiota signatures across substances beyond cocaine suggests that these adaptations may serve as candidate biomarkers for disorders characterized by loss of control. Such biomarkers could help refine diagnostic approaches and deepen our understanding of the complex interplay between reward systems, gut microbiota, and compulsive behaviors.

## Glossary

ABX: antibiotic
AN: anorexia nervosa
ASV: amplicon sequence variants
BED: binge eating disorder
BN: bulimia nervosa
CPP: conditioned place preference
CUD: cocaine use disorder
DSM-5: diagnostic and statistical manual of mental disorders
ED: eating disorder
FA: food addiction
FMT: fecal microbiota transplantation
HFD: high fat diet
HFHSD: high fat high sugar diet
HSD: high sugar diet
ICD-11: international classification of diseases
IVSA: intravenous self-administration
OTU: operational taxonomic unit
rRNA Seq: 16S ribosomal RNA sequencing
SCFA: short-chain fatty acid
SUD: substance use disorder
WT: wild type
